# Long‐term follow‐up results of pediatric and adolescent patients with localized Ewing sarcoma treated based on stratified modalities

**DOI:** 10.1002/cam4.5703

**Published:** 2023-02-21

**Authors:** Chenggong Zeng, Tingting Chen, Juan Wang, Feifei Sun, Junting Huang, Suying Lu, Jia Zhu, Yizhuo Zhang, Xiaofei Sun, Zijun Zhen

**Affiliations:** ^1^ State Key Laboratory of Oncology in South China Sun Yat‐sen University Cancer Center Guangzhou China; ^2^ Department of Pediatric Oncology Sun Yat‐sen University Cancer Center Guangzhou China; ^3^ Collaborative Innovation Center of Cancer Medicine Sun Yat‐sen University Cancer Center Guangzhou China

**Keywords:** chemotherapy, localized Ewing sarcoma, stratified treatment, survival

## Abstract

**Background:**

Compared with other pediatric tumors, little advances were achieved in studies on the stratified treatment in localized Ewing sarcoma. Most pediatric oncology groups treated Ewing sarcoma according to whether there was an existing metastasis, without involving more prognostic factors. In this study, patients with localized Ewing sarcoma were divided into resectable and unresectable groups at diagnosis and received chemotherapy with different intensity, for the purpose of achieving good efficacy, avoiding overtreatment and reducing unnecessary toxicity.

**Methods:**

A total of 143 patients with a median age of 10 years old diagnosed with localized Ewing sarcoma in this retrospective study were divided into two cohorts (Cohort 1, *n* = 42; Cohort 2, *n* = 101) and patients in Cohort 2 received chemotherapy with different intensity (Regimen 1, *n* = 52; Regimen 2, *n* = 49). Outcomes were analyzed using the Kaplan–Meier method to estimate event‐free survival (EFS) and overall survival (OS), and the curves were compared using the log‐rank test.

**Results:**

The 5‐year EFS and 5‐year OS for all the patients were 69.0% and 77.5%. The 5‐year EFS for Cohort 1 and Cohort 2 were 76.0% and 66.1% (*p* = 0.31), and the 5‐year OS were 83.0% and 75.1% (*p* = 0.30), respectively. In Cohort 2, the 5‐year EFS rate of patients treated with Regimen 2 was significantly higher than that of patients treated with Regimen 1 (74.5% vs. 58.3%, *p* = 0.03).

**Conclusions:**

According to whether a grossly complete resection was received at the time of diagnosis, localized Ewing sarcoma patients in this study were stratified into two groups and received different intensities of chemotherapy, which achieved good efficacy and avoided overtreatment and reduced unnecessary toxicity.

## Graphical abstract

This article revealed that localized Ewing sarcoma patiebts were stratified into two groups and received different intensities of chemotherapy, which achieving good efficacy.

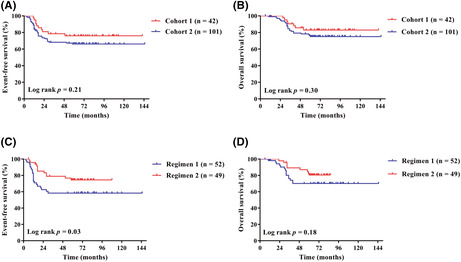

## 1 INTRODUCTION

Ewing sarcoma is an aggressive form of tumor that mainly occurs in children and adolescents, which includes classic Ewing's sarcoma, peripheral primitive neuroectodermal tumor (PNET), and Askin tumor. Ewing sarcoma can occur in bone or extraosseous soft tissue, accounting for approximately 3% of all childhood and adolescent cancers.^1^ Significant progress has been made for the treatment of Ewing sarcoma over the last 30 years. Only 10% of Ewing sarcoma patients achieved long‐term survival before the introduction of chemotherapy,^2^ while the 5‐year survival of patients with localized Ewing sarcoma has reached 70%–80% now.^3^

The types of treatments for Ewing sarcoma mainly included surgery, chemotherapy, and radiotherapy.^4^ For the chemotherapy in localized Ewing sarcoma, the North American intergroup Ewing sarcoma study INT‐0091 demonstrated that a regimen of vincristine, doxorubicin, and cyclophosphamide alternated with ifosfamide and etoposide (VDC/IE), delivered every 3 weeks for 17 courses, with a 5‐year progression‐free survival (PFS) reaching 69%.^5^ This regimen was later changed to be delivered every 2 weeks for 14 courses in COG AEWS 0031 study, and the 5‐year PFS was increased to 73%.^6^ More recently, the EURO‐E.W.I.N.G.99 trial confirmed that the event‐free survival of localized high‐risk Ewing sarcoma patients received high‐dose BuMel chemotherapy supported by blood autologous stem‐cell rescue for consolidation treatment was better than standard chemotherapy (vincristine, dactinomycin, and ifosfamide for seven courses).^7^ It suggested that increasing the intensity of chemotherapy may improve the prognosis of Ewing sarcoma, despite the fact that the same result has not been achieved in other studies.^4^, ^8^, ^9^

Compared with other pediatric tumors,^10^, ^11^, ^12^ little advances were achieved in studies on the stratified treatment in Ewing sarcoma. Most pediatric oncology groups only treated Ewing sarcoma patients according to whether there was an existing metastasis without involving in other prognostic factors.^5^, ^6^, ^13^, ^14^ Actually, there were many factors affecting the prognosis of Ewing sarcoma, among which tumor size at diagnosis has been confirmed closely related with long‐term survival by several studies.^15^, ^16^, ^17^, ^18^ However, for localized Ewing sarcoma, most pediatric oncology groups still use VDC/IE for 17 courses, and rarely adopted different intensities of chemotherapy according to the classification by tumor size at diagnosis.

Previous studies have concluded that Ewing sarcoma was highly aggressive and easily metastatic; therefore, it was recommended that all patients diagnosed with Ewing sarcoma should undergo neoadjuvant chemotherapy before surgery.^19^ However, in this study, due to the small size and off‐axial sites of tumors, some patients with localized Ewing sarcoma received radical surgery or not as first therapy, followed by adjuvant therapy including chemotherapy with or without radiotherapy. Because of good prognostic factors mentioned above, we adopted reduced‐intensity VDC/IE adjuvant chemotherapy, including reduction of partial drug dose and total courses compared with the INT‐0091 regimen. This study retrospectively analyzed the long‐term follow‐up outcomes of patients with initially resectable localized Ewing sarcoma and patients with unresectable tumors at diagnosis.

## 2 PATIENTS AND METHODS

### 2.1 Study design

Single institution retrospective study.

### 2.2 Patients

Patients had to be younger than 18 years old; diagnosed pathologically with classic Ewing's sarcoma, PNET, or Askin tumor; without metastasis; without definite contraindications to chemotherapy before treatment; and have not received anti‐tumor treatment before inclusion; and ever treated in Sun Yat‐sen University Cancer Center. Patients were divided into two cohorts, Cohort 1 was defined as patients with grossly complete tumor resection firstly, with no tumor residue or metastasis by imaging after surgery. Cohort 2 was defined as patients with unresectable tumor because of large tumor volume or axial tumor sites.

### 2.3 Diagnosis

All patients had received pathological biopsy as well as cytomorphology, immunohistochemistry with or without examination of EWS–FLI1 fusion before treatment. Pathological diagnosis of Ewing sarcoma required the presence of a small, blue, round‐cell tumor and positivity for CD99 and/or synaptophysin or chromogranin. Other round‐cell tumors were ruled out by immunohistochemistry with leucocyte common antigen, desmin, and myogenin. Computed tomography or magnetic resonance imaging was used to evaluate the extent of the local lesions. All patients underwent examination of metastasis, including bone scan, bone marrow cytology and pathology examination, and CT scan of the chest and abdomen and/or PET‐CT.

### 2.4 Chemotherapy

Regimen 1 was defined as VDC/IE delivered every 3 weeks for 8 courses. VDC was delivered on Day 1 of each cycle and it included vincristine (VCR) 1.5 mg/m^2^/day (maximal dose, 2 mg), doxorubicin (ADR) 50 mg/m^2^/day (Dexrazoxane for protection), and cyclophosphamide (CTX) 1000 mg/m^2^/day (followed by Mesna). IE was delivered on Days 1 through 5 of each cycle and it included ifosfamide (IFO) 1.5 g/m^2^/day (followed by Mesna) and etoposide (VP16) 100 mg/m^2^/day.

Regimen 2 was defined as dose‐intensified VDC/IE delivered every 3 weeks for 12 courses. The VDC was delivered on Day 1 of each cycle and it included VCR 1.5 mg/m^2^/day (maximal dose, 2 mg), ADR 60 mg/m^2^/day (Dexrazoxane for protection), and CTX 1200 mg/m^2^/day (followed by Mesna). IE was delivered on Days 1 through 5 of each cycle and it included IFO 1.8 g/m^2^/day (followed by Mesna) and VP16 100 mg/m^2^/day.

Between January 2002 and September 2012, Patients in Cohort 1 received Regimen 1 as an adjuvant chemotherapy after surgery. While patients in Cohort 2 received Regimen 1 as a neoadjuvant chemotherapy, and if it was effective, patients would receive with this regimen after local control. Between September 2012 and December 2016, patients in Cohort 1 were still treated with Regimen 1 as an adjuvant chemotherapy, while patients in Cohort 2 received Regimen 2 as induction chemotherapy and consolidation therapy.

### 2.5 Local control

Local control included surgery and radiotherapy. Patients in Cohort 1 received grossly complete resection first, followed by adjuvant chemotherapy. Grossly complete resection is defined as no tumor residual after surgery by CT or MRI. Radical resection is defined as extensive resection of the primary tumor, surrounding normal tissues and lymph node drainage area, and the surgical margin is negative. Excluding patients received radical resection, other patients received grossly complete resection is defined as non‐radical resection. In addition, then if they received radiotherapy, the dose would be 45–55 Gy with VI regimen as a concurrent chemotherapy for two courses. The VI regimen was delivered on Days 1 through 5 of each cycle for every 3 weeks and it included VCR 1.5 mg/m^2^/day (maximal dose, 2 mg) and irinotecan 50 mg/m^2^/day. Patients in Cohort 2 received induction chemotherapy first, followed by surgery and radiotherapy. The dose of radiotherapy was 45–55 Gy. During radiotherapy, VI regimen was used as concurrent chemotherapy for two courses.

### 2.6 Evaluation of efficacy and toxicity

The efficacy was evaluated according to the World Health Organization efficacy evaluation criteria of solid tumors.^20^ Chemotherapeutic toxicity was evaluated according to the evaluation criteria of the National Cancer Institute.^21^

### 2.7 Statistical methods

The primary study endpoints were event‐free survival (EFS) and overall survival (OS). The EFS was defined as the time from entry onto the study until the occurrence of an adverse event (disease progression, second malignant neoplasm, or death) or until the last contact with the patient, whichever came first. The OS was defined as the time from entry into the study until death or until last contact with the patient, whichever came first. The OS and EFS were estimated using the Kaplan–Meier method, and the log‐rank statistic was used to compare the risk of an adverse event between groups. The HRs and significance associated with patient characteristics at enrollment were assessed in a proportional hazards regression model. SPSS 25.0 and GraphPad 8.0 were used for statistical calculations.

## 3 RESULTS

### 3.1 Patient characteristics

Between January 2002 and December 2016, 143 patients were enrolled in this study with 42 patients in Cohort 1 and 101 patients in Cohort 2. The ratio of male to female was 1.8:1, and the median age at diagnosis was 10 years old (1 month to 18 years old). The remaining detailed features are shown in Table 1.

**TABLE 1:** Patient characteristics.

		Cohort 2			
Characteristics	Cohort 1, *n* (%)	Regimen 1, *n* (%)	Regimen 2, *n* (%)	Total, *n* (%)	*p*‐Value^a^
Gender					0.52
Male	22 (52)	38 (73)	32 (65)	92 (64)	
Female	20 (48)	14 (27)	17 (35)	51 (36)	
Age (years)					0.07
≤13	28 (67)	39 (75)	44 (90)	111 (78)	
>13	14 (33)	13 (25)	5 (10)	32 (22)	
Pathologic type					0.22
Classic Ewing's sarcoma	20 (48)	28 (54)	33 (67)	81 (57)	
PNET	20 (48)	23 (44)	16 (33)	59 (41)	
Askin tumor	2 (4)	1 (2)	0 (0)	3 (2)	
Primary site					0.09
Truncus	6 (14)	11 (21)	10 (20)	27 (19)	
Head, neck	12 (29)	14 (27)	17 (35)	43 (30)	
Limbs	17 (40)	24 (46)	13 (27)	54 (38)	
Retroperitoneal	7 (17)	3 (6)	9 (18)	19 (13)	
Tumor volume (mL)					0.67
≤200	36 (86)	36 (69)	36 (73)	108 (75)	
>200	6 (14)	16 (31)	13 (27)	35 (25)	
Lactate dehydrogenase (U/L)					0.74
≤500	38 (90)	46 (88)	45 (92)	129 (90)	
>500	4 (10)	6 (12)	4 (8)	14 (10)	

^a^ *p*‐Values for the comparison of frequencies for Regimen 1 and Regimen 2 were calculated by chi‐square tests.

### 3.2 Treatment stratification

**FIGURE 1:**
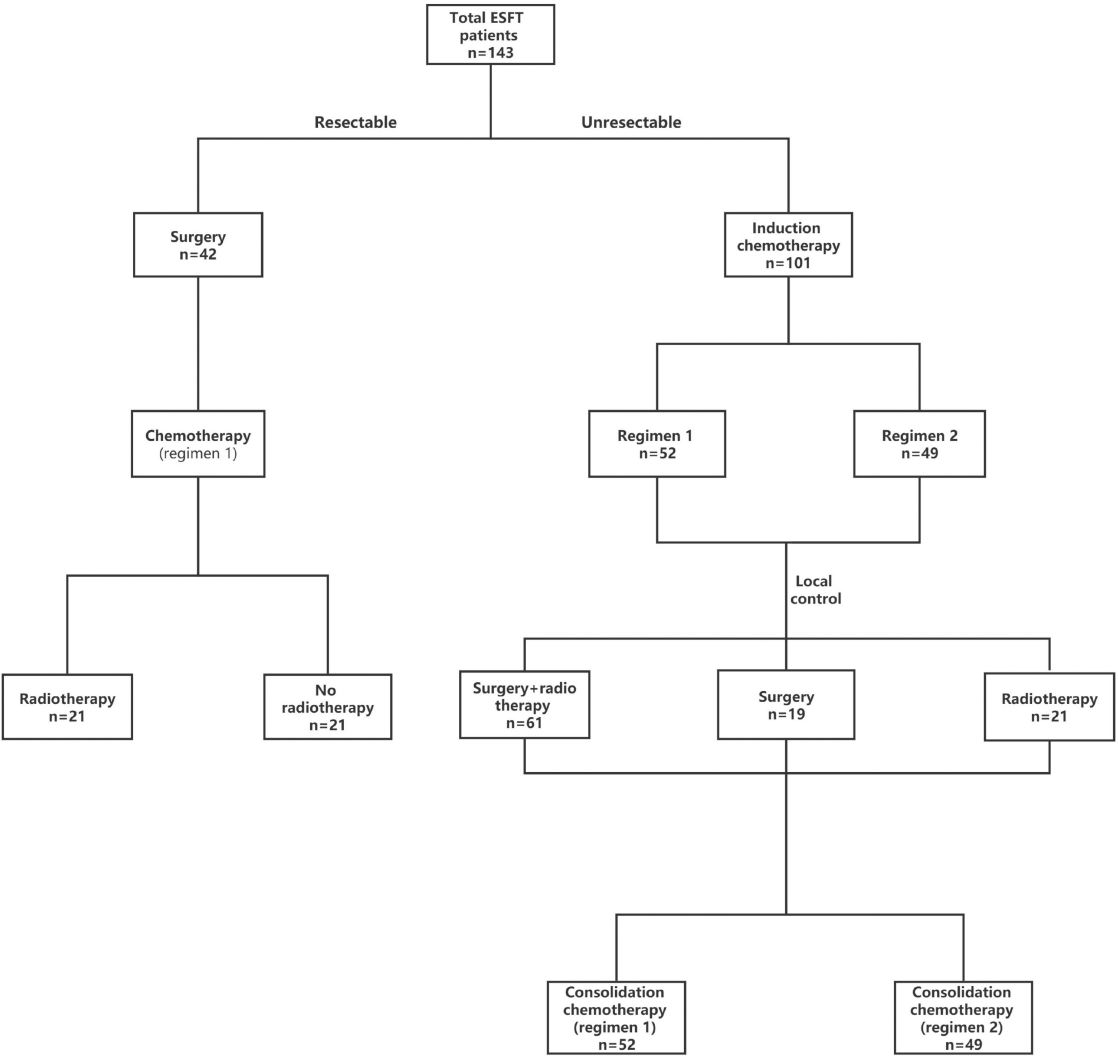
Treatment process of the whole group of patients. Different treatment modes were adopted according to whether a grossly complete resection was received at diagnosis. Resectable means that the tumor was grossly removed at diagnosis, without tumor residue and metastasis detected on imaging examination after surgery.

In Cohort 1, all 42 patients underwent grossly complete resection at the time of diagnosis, among which 12 patients underwent radical resection at the time of diagnosis, while 30 patients underwent non‐radical surgery. After surgery, all patients in Cohort 1 received Regimen 1 for eight courses, among which 21 patients received radiotherapy subsequently. In Cohort 2, all 101 patients were diagnosed by pathological biopsy, and 52 patients received Regimen 1 for induction chemotherapy and consolidation chemotherapy, while 49 patients received Regimen 2. The local control in Cohort 2 was taken according to the specific situation, among which 19 patients received surgery, 21 received radiotherapy, and 61 received surgery combined with radiotherapy. See Figure 1 for the specific process.

### 3.3 Efficacy and side effects of chemotherapy

Objective response rate (ORR) was defined as the proportion of patients whose tumor volume decreased by 30% and could be maintained for more than 4 weeks, which was to say the sum of complete remission (CR) and partial remission (PR). All of the 101 patients in Cohort 2 had evaluable lesions; 52 patients received Regimen 1 with ORR of 88.5%, and 49 patients received Regimen 2 with ORR of 93.9%. As for the patients who received Regimen 1 and Regimen 2, the incidence of grade III‐IV myelosuppression was 29.8% and 38.8% (*p* = 0.35), and the incidence of infection was 14.9% and 18.4% (*p* = 0.64), respectively. By the end of follow‐up, there was no cardiotoxicity, second malignancy, or treatment‐related deaths observed.

### 3.4 Survival

**FIGURE 2:**
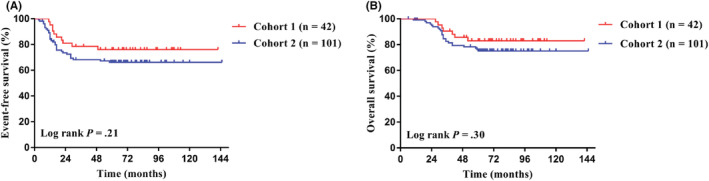
Comparison of long‐term survival of localized Ewing sarcoma patients between Cohort 1 and Cohort 2. (A) There was no significant difference in the 5‐year overall survival (OS) between the two groups. (B) There was no significant difference in the 5‐year OS between the two groups.

**FIGURE 3:**
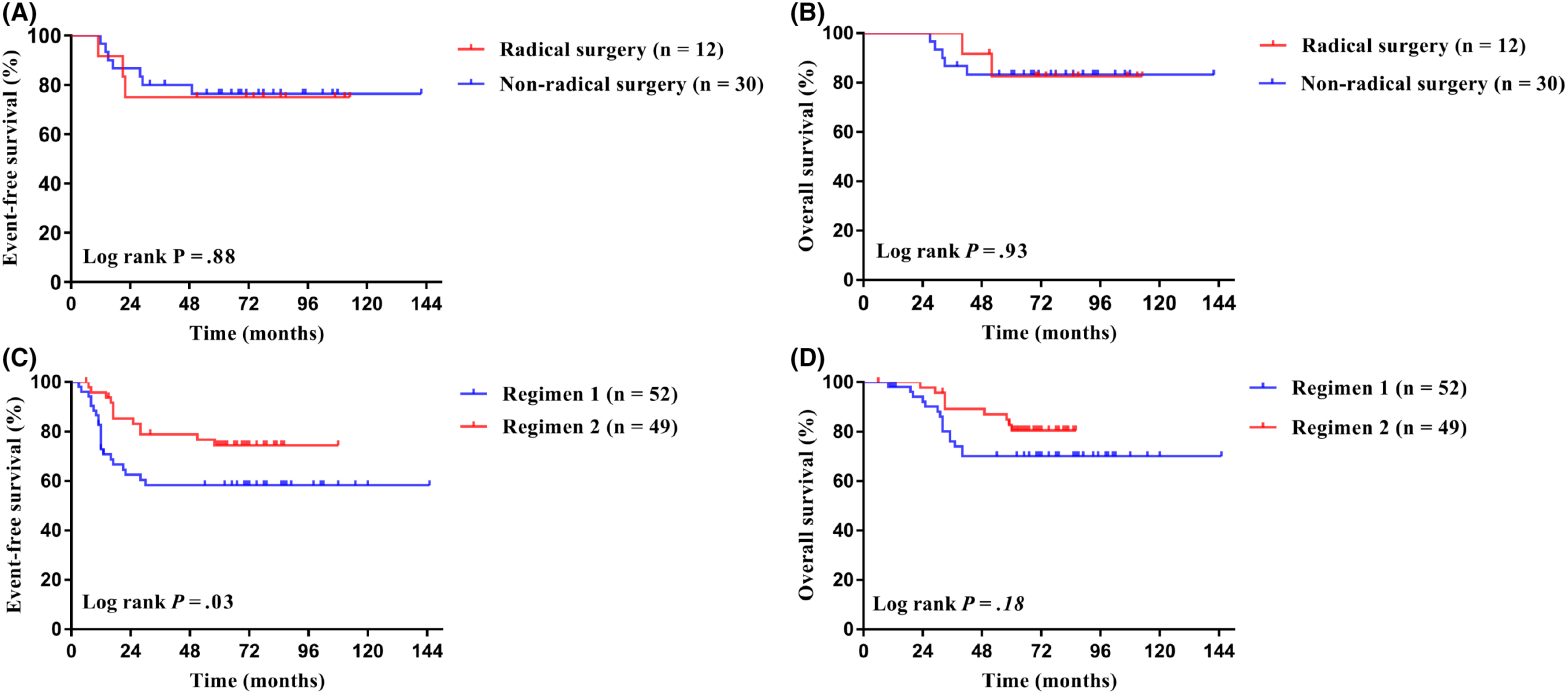
Subgroup analysis of Cohort 1 and Cohort 2. (A) There was no difference in the 5‐year event‐free survival (EFS) in patients with or without radical resection in Cohort 1. (B) There was no difference in the 5‐year OS between patients with or without radical resection in Cohort 1. (C) The 5‐year EFS in Cohort 2 treated with Regimen 2 was better than Regimen 1. (D) There was no difference in the 5‐year OS between patients using Regimen 1 and 2 in Cohort 2.

The last follow‐up date was December 31, 2021, with a median follow‐up time of 68 months (6–145 months). The 5‐year EFS and 5‐year OS of the whole group were 69.0% (95% CI, 60.6%–76%) and 77.5% (95% CI, 69.6%–83.6%). The 5‐year EFS for Cohort 1 and Cohort 2 were 76.0% and 66.1% (95% CI, 60.0%–86.3% for Cohort 1; 95% CI, 55.6%–74.6% for Cohort 2; *p* = 0.31; Figure 2A), and the 5‐year OS were 83.0% and 75.1% (95% CI, 67.5%–91.4% for Cohort 1; 95% CI, 65.2%–82.6% for Cohort 2; *p* = 0.30; Figure 2B), respectively. In Cohort 1, the 5‐year EFS for patients undergoing radical surgery and non‐radical surgery were 75.0% and 76.4% (95% CI, 41.0%–91.2% for radical surgery; 95% CI, 56.7%–88.0% for non‐radical surgery; *p* = 0.88; Figure 3A), and the 5‐year OS were 82.5% and 83.2% (95% CI, 46.1%–95.3% for radical surgery; 95% CI, 64.2%–92.6% for non‐radical surgery; *p* = 0.93; Figure 3B), respectively. In Cohort 2, the 5‐year EFS for patients treated with Regimen 2 was significantly higher than that of patients treated with Regimen 1, specifically 74.5% versus 58.3% (95% CI, 59.4%–84.7% for Regimen 2; 95% CI, 43.4%–70.5% for Regimen 1; *p* = 0.03; Figure 3C), and the 5‐year OS were 80.9% and 69.5% (95% CI, 65.8%–89.4% for Regimen 2; 95% CI, 55.4%–80.8% for Regimen 1; *p* = 0.11; Figure 3D), respectively.

**FIGURE 4:**
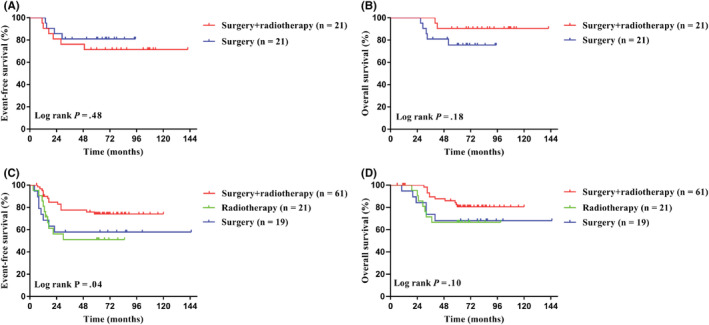
Effect of local control on the long‐term survival for patients with localized ESFT. (A) There was no difference in the 5‐year event‐free survival (EFS) between patients with surgery + radiotherapy and surgery alone in Cohort 1. (B) There was no difference in the 5‐year overall survival (OS) between surgery + radiotherapy and surgery alone in Cohort 1. (C) The 5‐year EFS in Cohort 2 treated with surgery + radiotherapy was better than surgery alone and (or) radiotherapy alone. (D) There was no difference in the 5‐year OS in Cohort 2 treated with surgery + radiotherapy, surgery alone, and radiotherapy alone.

For the local control, among Cohort 1, the 5‐year EFS for 21 patients with surgery and 21 patients with surgery combined with radiotherapy were 81.0% and 71.4% (95% CI, 56.9%–92.4% for surgery alone; 95% CI, 47.1%–86.0% for surgery combined with radiotherapy; *p* = 0.48; Figure 4A), and the 5‐year OS were 75.5% and 90.5% (95% CI, 50.7%–89.0% for surgery alone; 95% CI, 67.0%–97.6% for surgery combined with radiotherapy; *p* = 0.18; Figure 4B), respectively. Among Cohort 2, the 5‐year EFS for patients with surgery combined with radiotherapy was significantly higher than using surgery or radiotherapy alone, specifically 74.0% versus 57.9% versus 51.0% (95% CI, 60.6%–83.4% for surgery combined with radiotherapy; 95% CI, 33.2%–76.3% for surgery alone; 95% CI, 28.0%–70.0% for radiotherapy; *p* = 0.04; Figure 4C), and the 5‐year OS were 80.6%, 66.7%, and 68.0% (95% CI, 67.7%–88.8% for surgery combined with radiotherapy; 95% CI, 42.1%–84.2% for surgery alone; 95% CI, 42.6%–82.5% for radiotherapy; *p* = 0.10, Figure 4D), respectively.

### 3.5 Prognostic factors

We performed a logistic regression analysis on age, gender, pathological type, tumor volume, primary tumor site, and value of serum lactate dehydrogenase (LDH). We found that patients with Ewing sarcoma with tumor volume >200 mL, LDH >500 U/L at diagnosis and tumor primary site on the truncus had a poor prognosis. See Table 2 for more details.

**TABLE 2:** Analysis of prognosis for localized Ewing sarcoma under different subgroups.

		Cohort 1						Cohort 2					
		Regimen 1						Regimen 1			Regimen 2		
		EFS			OS			EFS			OS		
Variable	Subgroup	HR	5‐year estimate(%)	*p*	HR	5‐year estimate (%)	*p*	HR	5‐year estimate (%)	*p*	HR	5‐year estimate (%)	*p*
Age (years)	≤13	0.6	81.1	0.40	0.7	84.8	0.60	0.5	70.2	0.03	0.4	78.8	0.04
	>13	1.7	71.4		1.4	78.6		2.3	45.5		2.4	56.3	
Gender	Female	0.5	85.0	0.25	1.5	80.0	0.62	1.1	63.4	0.85	1.3	70.0	0.50
	Male	2.2	67.9		0.7	86.1		0.9	67.5		0.8	77.4	
Pathologic type	Classic Ewing's sarcoma	0.2	89.7	0.03	0.2	94.4	0.04	0.5	73.4	0.08	0.6	80.1	0.15
	Non‐classic Ewing's sarcoma	4.5	63.3		6.2	72.7		1.8	56.1		1.8	68.2	
Tumor Volume (mL)	≤200	0.6	77.5	0.50	1.1	83.1	0.94	0.4	72.6	0.01	0.3	81.4	0.005
	>200	1.7	66.7		0.9	83.3		2.4	42.9		3.0	52.4	
LDH (U/L)	≤500	0.9	76.0	0.98	0.6	83.9	0.65	0.3	70.3	0.005	0.4	78.0	0.07
	>500	1.0	75.0		1.6	75.0		3.1	30.0		2.4	50.0	
Primary site	Non‐truncus	0.4	79.7	0.19	0.4	85.3	0.30	0.7	67.6	0.40	0.5	79.1	0.10
	Truncus	2.4	57.1		2.3	71.4		1.3	60.0		2.0	60.0	

Abbreviations: EFS, event‐free survival; HR, hazard ratio; LDH, lactate dehydrogenase; OS, overall survival.

## 4 DISCUSSION

Stratified treatment was used in children and adolescents with localized Ewing sarcoma, with the aim of improving the level of individualized treatment. For patients with tumor grossly complete resected firstly and without tumor residue or metastasis after postoperative imaging, we used VDC/IE for eight courses with or without radiotherapy, and ultimately achieved good results, in which the 5‐year EFS and OS were 76.0% and 83.0%. The results were similar to those of most international clinical studies on localized Ewing sarcoma.^5^, ^6^, ^7^ After nearly 40 years of evolution, the chemotherapy courses of Ewing sarcoma have been gradually shortened, but the widely used INT‐0091 regimen still takes 17 courses, which up to 1 year.^5^ As for solid tumors, whether localized Ewing sarcoma requires long periods of chemotherapy is unclear, and no randomized controlled clinical study has determined its ideal duration of chemotherapy.

For patients with tumor grossly complete resected because of small volume and off‐axial sites at the time of diagnosis, we reduced the intensity of chemotherapy but did not decrease efficacy. The chemotherapeutic drug compositions of regimen in this study were the same as the INT‐0091 regimen, but compared to it,^5^ the single dose of most of the drugs were decreased in Regimen 1, and the cumulative dose decreased to half or less (Table 3). Our findings suggested that prolonged chemotherapy to 17 courses in these patients in Cohort 1 was significantly overtreated. Moreover, the increasing in the cumulative dose of chemotherapy brought much long‐term toxicity to patients, including second malignancy,^22^ cardiac toxicity,^23^ chronic lung disease,^24^, ^25^ and infertility.^26^ Long‐term chemotherapy courses of up to 1 year also brought great psychological and economic burden to patients, and even affected their compliance to medical treatment.

**TABLE 3:** Comparison of drug dose for localized Ewing sarcoma in this study.

	Dose per course				Cumulative dose			
Drugs	INT‐0091 regimen	Regimen 1	Regimen 2	VI regimen	INT‐0091 regimen	Regimen 1	Regimen 2	VI regimen
Vincristine (mg/m^2^)	2	1.5	1.5	1.5	18	6	9	3
Doxorubicin (mg/m^2^)	75	50	60	0	375	200	360	0
Cyclophosphamide (g/m^2^)	1.2	1	1.2	0	10.8	4	7.2	0
Ifosfamide (g/m^2^)	1.8 × 5	1.5 × 5	1.8 × 5	0	72	30	54	0
Etoposide (mg/m^2^)	100 × 5	100 × 5	100 × 5	0	4000	2000	3000	0
Irinotecan (mg/m^2^)	0	0	0	50 × 5	0	0	0	500

*Note*: VI regimen, concurrent chemotherapy during radiotherapy which includes vincristine and irinotecan.

However, locally advanced patients with inoperable tumor received Regimen 1 combined with surgery and/or radiotherapy, resulting in poor survival with 5‐year EFS and OS reaching 58.3% and 69.5%. Without advancement in the local control methods, we believed that the main reason for the poor prognosis of these patients was the insufficient intensity of chemotherapy. Some previous studies also shown that increasing chemotherapy intensity increased the efficacy of localized Ewing sarcoma.^5^, ^6^, ^7^ Because of that, for the locally advanced Ewing sarcoma patients, the single dose of cyclophosphamide, doxorubicin, and ifosfamide was increased since 2012, and the total chemotherapy courses were also increased to 12, significantly improving the prognosis of these patients. Although the intensity of Regimen 2 was higher than Regimen 1, the single dose of doxorubicin and the cumulative dose of each chemotherapy agent were still lower than the INT‐0091 regimen.

Nonetheless, some studies have shown that increasing chemotherapy intensity did not improve Ewing sarcoma efficacy.^8^, ^9^, ^27^ Chemotherapy occupied only one part in the multidisciplinary comprehensive treatment of Ewing sarcoma, and local control modalities had an important impact on long‐term survival. This study also explored the prognosis of different local control modalities and found that surgery combined with radiotherapy was significantly better than surgery alone or radiotherapy alone. However, the local control modality appropriate for Ewing sarcoma remained controversial, and some studies believed that radical resection was difficult to perform for most large‐volume or midline tumors, and additional radiotherapy can improve survival.^28^ However, when the patients were too young or the tumor occurred in the abdominal cavity or pleural areas, the dose and range of radiotherapy were limited. At this time, surgery was a better choice.^29^ It can be seen that many factors affect the choice of local control, and local control further affects the prognosis.

For the factors affecting the prognosis of Ewing sarcoma, besides the choice of treatment methods, previous studies have identified several other adverse prognostic factors, including metastasis, large tumor size,^15^, ^16^, ^17^ primary tumor site on the truncus,^15^, ^30^, ^31^ age >13 years old,^15^, ^17^, ^19^ male gender,^32^ poor response to induction chemotherapy,^33^, ^34^ etc. This study also confirmed that the tumor volume larger than 200 mL and the primary tumor site on the truncus were poor prognostic factors. We also revealed that the LDH twofold higher than the normal value was also associated with poor prognosis, which had certain guiding significance for clinical treatment. We did not find the age and gender affecting the prognosis, which may be related to the small number of cases.

Previous studies have concluded that Ewing sarcoma was a highly aggressive and easily metastatic tumor; thus, it was recommended that all patients should undergo neoadjuvant chemotherapy before surgery.^29^ However, in this study, patients in Cohort 1 underwent surgery after diagnosis followed by adjuvant therapy, and achieved good results. Moreover, the subgroup analysis found that local control methods did not affect the prognosis of these patients. The reason may be that these patients have much good prognostic factors, including superficial and off‐axial sites, early diagnosis, small tumor volume and receiving effective treatment before metastasis. Because Ewing sarcoma was highly sensitive to radiotherapy and chemotherapy, although maybe there are minimal residual lesions after surgery, we can still eliminate them through a timely and positive adjuvant therapy. Therefore, we suggest that the treatment model of Ewing sarcoma can be diversified and initially assessed by surgeon at diagnosis.

The limitation of this study was that it was a retrospective, single‐centre and non‐randomized controlled study with a small sample size. And this study has spanned 15 years, which may affect the results. On the other hand, considering the prognosis for adult with Ewing sarcoma seems to be inferior to younger patients,^17^ patients enrolled in this study were totally children and adolescents, which may influence the comparison. Although the EFS in this study achieved statistical significance, the OS missed conventional statistical significance. The reason may be that the treatment methods for patients who relapsed in this study was not uniform, and some patients even abandoned further treatment for various reasons, which may affect the comparability of OS between groups.

In this study, patients with localized Ewing sarcoma were divided into resectable and unresectable groups at diagnosis and received different intensity of regimen, achieving good efficacy and avoiding overtreatment and reducing unnecessary toxicity. Strategies of stratified treatment for localized Ewing sarcoma have been gradually adopted by multiple research organizations and will benefit more patients.^4^, ^35^

## 5 CONCLUSIONS

According to whether a grossly complete resection was received at the time of diagnosis, localized Ewing sarcoma patients in this study were stratified into two groups and received different intensities of chemotherapy, which achieved good efficacy and avoided overtreatment and reduced unnecessary toxicity. The study also proved that increasing the intensity of chemotherapy can improve the prognosis of locally advanced Ewing sarcoma patients. While comparing with the chemotherapy regimen in INT‐0091 trial for localized Ewing sarcoma which includes 17 courses, the dose of chemotherapeutic drugs was still lower.

## AUTHOR CONTRIBUTIONS

**Chenggong Zeng:** Conceptualization (equal); data curation (equal); formal analysis (equal); methodology (equal); software (equal); writing – original draft (lead); writing – review and editing (equal). **Tingting Chen:** Data curation (equal); formal analysis (equal); methodology (equal); validation (equal); writing – review and editing (equal). **Juan Wang:** Investigation (equal). **Feifei Sun:** Investigation (equal). **Junting Huang:** Investigation (equal). **Suying Lu:** Investigation (equal). **Jia Zhu:** Investigation (equal). **Yizhuo Zhang:** Investigation (equal); supervision (equal). **Xiaofei Sun:** Conceptualization (equal); methodology (equal); project administration (equal); supervision (equal); validation (equal); writing – review and editing (equal). **Zi‐jun Zhen:** Conceptualization (equal); methodology (equal); project administration (equal); supervision (equal); validation (equal); writing – review and editing (equal).

## FUNDING INFORMATION

The authors made no disclosures.

## CONFLICT OF INTEREST STATEMENT

The authors have no conflict of interest to declare.

## ETHICAL STATEMENT

The study was conducted in accordance with the Declaration of Helsinki, and approved by the Ethical Committee of Sun Yat‐sen University Cancer Center (approval number B2022‐079‐01) prior to the collection of all patient information from the electronic medical record. The requirement for exemptions from informed consent for this study was waived owing to the retrospective design of this study. The privacy and identity information of the patients are protected.

## Data Availability

The data that support the findings of this study are available from the corresponding author upon reasonable request. And the data were uploaded in Research Data Deposit at https://www.researchdata.org.cn, reference number RDDA2022960205 (accessed on 12 April 2022). The data are not publicly available due to privacy or ethical restrictions.
